# Long-term incidence of cardiovascular outcomes in the middle-aged and elderly with different patterns of physical activity: Tehran lipid and glucose study

**DOI:** 10.1186/s12889-020-09747-6

**Published:** 2020-11-04

**Authors:** Parisa Naseri, Parisa Amiri, Hasti Masihay-Akbar, Sara Jalali-Farahani, Davood Khalili, Fereidoun Azizi

**Affiliations:** 1 Research Center for Social Determinants of Health, Research Institute for Endocrine Sciences, Shahid Beheshti University of Medical Sciences, P.O.Box: 19395-4763, Tehran, IR Iran; 2 Prevention of Metabolic Disorders Research Center, Research Institute for Endocrine Sciences, Shahid Beheshti University of Medical Sciences, Tehran, Iran; 3 Endocrine Research Center, Research Institute for Endocrine Sciences, Shahid Beheshti University of Medical Sciences, Tehran, Iran

**Keywords:** Cardiovascular disease, Coronary heart disease, Physical activity, Eastern-Mediterranean

## Abstract

**Background:**

Following the global upward trend of cardiovascular diseases (CVD/CHD), much attention has been paid to lifestyle behaviors such as physical activity (PA). However, most of previous studies were conducted in developed countries and with just one measurement of physical activity. The aim of the current study is to assess the effect of changes in the PA on the incidence of CVD/CHD in middle-aged and older men and women in an Eastern-Mediterranean population, over a decade follow-up.

**Methods:**

This study has been conducted within the framework of the Tehran Lipid and Glucose Study (TLGS) including 4073 (57% women) participants without CVD/CHD at baseline. The participants were followed up for an average period of 12 years. The Iranian version of Modified Activity Questionnaire (MAQ) was used to measure PA at baseline and at the closest follow-up to the outcome. Subsequently, participants were categorized as “compliers”, “non-compliers”, “adopters” and “relapsers”, based on their adherence to the PA guideline recommendations. All analysis has been conducted in two separate age groups including middle-aged and elderly in both men and women. The effect of PA patterns on incidence of CVD/CHD was investigated using Cox proportional hazard model. Variables including marital status, job status, education, smoking, and family history of CVD/CHD were adjusted in the models.

**Results:**

Adherence to guideline recommendation increased from 63.5 to 66.6% between the two measurements. At the second measurement of PA, the percentages of compliers, non-compliers, adopters and relapsers were 48.4, 18.3, 18.2, and 15.1%, respectively. In fully adjusted models, HRs of CVD and CHD for men aged 40–60 years in the complier group were 0.58 (95% CI: 0.38–0.87, *P* = 0.008) and 0.58 (95% CI: 0.38–0.89, *P* = 0.01), respectively. HRs of CVD and CHD for men aged 40–60 years in adopter group were 0.61 (95% CI: 0.38–0.96, *P* = 0.03) and 0.60 (95% CI: 0.37–0.97, *P* = 0.04) respectively. The corresponding values were not significant in women.

**Conclusions:**

Adhering to established PA recommendations have a protective effect on the incidence of CVD/CHD among middle-aged men; findings which need to be considered in reducing cardiovascular outcomes in this population.

## Background

Cardiovascular diseases (CVDs) are the most important causes of mortality worldwide and it is estimated that the overall number of deaths caused by CVDs will be increased to 20 million by 2030 [1]. According to the Global Burden of Disease study, in Europe, coronary heart disease (CHD) as the most common type of CVDs accounts for 20% of overall mortality [2]. Similarly, high prevalence of CVD and CHD is reported in the Middle East. Currently, 46% of all deaths and 20–23% of the burden of diseases in Iran are caused by CVDs [3, 4]. It is estimated that two billion people will be older than 60 years by the year 2050 [5]. In developed countries, about 75% of deaths in individuals older than 65 years are caused by CVDs and cancers [6]. Considering this rapid growth of aging in the world, prevention of cardiovascular outcomes in the elderly seems a global necessity.

It is well-documented that the development of CVDs is associated with lifestyle [7]. Findings from 11 European countries, indicated the importance of lifestyle factors such as alcohol use, smoking status and physical activity (PA) in the association with CVD, CHD, cancers and all-cause mortality in the late adulthood [8]. Accordingly, population-wide surveillance data indicate that physical inactivity is more prevalent in older adults and having suitable levels of physical activity could improve health status in middle- and older-aged American women [9]. More evidence emphasized physical inactivity as a major predictor of survival time in older (aged 65–74 years) than in middle-aged persons (aged 45–54 years) [10]. In Iran, although evidence shows that a healthy lifestyle is an effective strategy to healthy aging, many of Iranian older adults do not accept health-promoting behaviors including qualified physical activity in their life [11]. In this regard, findings of a national survey in Iran showed that 40.0% of the population has low level of physical activity. Approximately 15% of Iranian adults do not have any physical activity in any of domains including physical activity at work, commuting and recreation [12].

The importance of an active lifestyle in reducing mortality risks [13–18] and prevention of CVDs [19, 20] is well known in the existing literature. Nevertheless, the majority of previous studies measured physical activity at one time point, hence failed to address the complexity and dynamic nature of this behavior [21–25]. The few studies which consider the patterns of physical activity changes over time, are mostly conducted in developed countries [20, 26–30]. Moreover, a large number of studies on the health effects of physical activity only considered men, and data on older women is sparse [24, 25, 31–33]. Considering the methodological differences in the previous studies, there is still inconsistency in changes of PA over-time and how they relate to CVD incidence, particularly among women and less-developed countries. In Iran, existing evidence regarding the association between physical activity and cardiovascular outcomes limited to cross-sectional results on specific populations living in the north and central parts of the country [34, 35]. To obtain a more reliable estimate of the mentioned association, longitudinal investigations would be beneficial. Hence, as one of the first efforts, the current study aimed to investigate the long-term incidence of cardiovascular outcomes in men and women with the changes of physical activity over a decade follow-up, in two age groups of middle-aged and elderly.

## Methods

### Study design and population

The Tehran Lipid and Glucose Study (TLGS) is an ongoing population-based cohort, designed to determine the prevalence of non-communicable diseases and their risk factors, conducted on a representative sample of residents of district 13 of Tehran. The design of the TLGS included two components: first phase, a cross-sectional study to assess prevalence of cardiovascular risk factors (1999–2001); and prospective ongoing follow-up examinations which have been continued every 3 years. Details on the rationale, sampling and data collection of the TLGS have been published elsewhere [36].

The current longitudinal analysis has been conducted on data from the second (2002–2004) to fifth (2011–2013) phases of the TLGS. A total of 4073 (57% women) participants without CVD and CHD at baseline (phase 2) who had complete sets of data were followed up for an average period of 12 years. The mean of survival time for CVD and CHD was 137.85 and 139.64 months respectively. Informed written consent was obtained from all participants. The ethics committee of the Research Institute for Endocrine Sciences, Shahid Beheshti University of Medical Sciences, approved the design of the TLGS study.

### Physical activity measurements

Physical activity, including leisure time and occupational activities, was assessed using reliable and validated Iranian version of Modifiable Activity Questionnaire (MAQ) [37]. Participants reported their physical activities as well as the frequencies and duration for each activity over the past 12 months. Total numbers of minutes/year for all leisure time physical activities were summed up and then divided by 52 to estimate total leisure time physical activities in minutes/week. Metabolic equivalent (MET) of total leisure time physical activity for each person was then calculated by multiplying the number of minutes/week of each leisure time activity to its MET. One MET is set at 3.5 ml of oxygen consumed per kg of body weight per minute and represents the resting metabolic rate. The calculation of MET-minutes/week is summarized as MET-minutes/week = (MET × months/year × sessions/month × minutes/session)/ 52. Then, MET-minutes/week was multiplied by weight of each person.

Employed persons were asked to indicate how many hours a week they usually worked. According to the questionnaire, individuals had to identify the number of months and hours they participated in physical activity at work (standing, housework, work activities) over the past year. The assessment of occupational activity was based on summing up the number of hours per week of light, moderate and vigorous intensity activities and then multiplying by 60 to express minutes per week of occupational activity over the past year. Finally, occupational (MET-minutes/week) activity was calculated by multiplying the number of minutes per week of each of the three categories of occupational activity by MET values. A total physical activity estimate was reached by adding leisure time physical activity to occupational activity and then categorized as low (< 600 MET.min/week), moderate (600–2999 MET.min/week), and high (≥ 3000 MET.min/week). Recommended level of physical activity was set as at least 150 min of moderate-intensity aerobic physical activity or ≥ 600 MET/minutes throughout the week. The participants who were moderately or highly active met the recommended physical activity, while those categorized as low, did not meet the guidelines [38].

Subsequently, in order to reflect the pattern of physical activity over time, a new classification was constructed using measurements of physical activity at two time points (baseline and the closest measurement before the event). This pattern showed the degree to which participants adhered to guideline recommendations over time. “Compliers” included those who met the recommendations at both measurements. In contrast “Non-compliers” were those not reaching the recommended levels at both measurements. Participants who were inadequately active at baseline but took up the recommendations at the second measurement, were named “Adopters”. Those who acted contrary to the previous group were named “Relapsers”.

### Health outcomes

Details of the definitions and analysis of outcome data have been described before [39]. In the current study, CHD was defined as cases of 1) definite myocardial infarction diagnosed by ECG and biomarkers, 2) probable myocardial infarction (positive ECG findings plus cardiac symptoms or signs but biomarkers showing negative or equivocal results), 3) unstable angina pectoris (new cardiac symptoms or changing symptom patterns and positive ECG findings with normal biomarkers), 4) angiographic-proven CHD, and 5) CHD death. CVD was defined as any measures of CHD events, plus stroke or cerebrovascular events.

### Covariates

Education level was categorized based on the study years as primary/illiterate (0–6 years), secondary/diploma (6–12 years) and higher (more than 12 years). Marital status was considered in three groups including single, married, and divorced/widowed. Smoking habits were categorized into smokers, and non-smokers. Participants were classified as employed and unemployed based on whether they have outcome or not.

### Statistical analysis

Participant characteristics were summarized as mean ± standard deviation and frequencies (percentages) for continuous and categorical variables, respectively in sex- age categories. In both genders, baseline characteristics were compared between age categories using independent sample T-tests for continuous variables and chi-square tests for categorical variables. Additionally, baseline characteristics of respondents and non-respondents, who were loss-to-follow up during the study period (*n* = 68) were compared by Student’s *t* test for continuous variables and the Chi-square test for categorical variables (data not shown). Cox proportional hazards (PH) models were fitted to evaluate the association of physical activity level with CVD and CHD outcomes, separately. For each outcomes, follow up duration was defined as the period between entrance to study and the end point of study; end point was considered as the first CVD and CHD outcome or censoring, which was defined as leaving the residence area, loss to follow up, non CVD and CHD death or end of follow-up.

The appropriateness of the proportionality assumption in the Cox model was assessed graphically and using Schoenfeld’s global test of residuals. All proportionality assumptions were generally appropriate.

The gender- and age-specific Hazard Ratios (HRs) and their 95% confidence intervals (CIs) for CVD and CHD outcomes were estimated in four different models. The first model was unadjusted (univariate model). The second model was adjusted for job status and education. In the third model smoking status was added and the fourth model was further adjusted for family history of CVD and CHD. In all models, the non-complier group was considered the referent. Statistical analyses were performed using STATA version 14 [40]. *P* < 0.05 was considered statistically significant.

## Results

A total of 4073 participants (1751 (43%) men and 2322 (57%) women) without CVD and CHD, aged ≥40 years were included in the current study. Baseline characteristics of the study population according gender and age groups are presented in Table 1. Smoking was not statistically different in age groups for women. However, other covariates including education, marital status, job status, and family history of CVD or CHD differed between age categories in both men and women (*P* < 0.05). Adherence to the physical activity guidelines increased from 63.5 to 66.6% between the two measurements. At the second measurement of physical activity, the percentages of compliers, non-compliers, adopters and relapsers were 48.4, 18.3, 18.2, and 15.1%, respectively.

**Table 1:** General characteristics of participants according to sex at baseline

Age groups	Male			Female		
	40–60	> 60	*P*- value	40–60	> 60	*P*-value
**Education**			< 0.001			< 0.001
Illiterate/primary	463 (39.1)	449 (79.0)		1190 (66.6)	517 (96.6)	
Secondary/diploma	482 (40.7)	73 (12.9)		487 (27.3)	13 (2.4)	
Higher	238 (20.1)	46 (8.1)		110 (6.2)	5 (0.9)	
**Marital status**			0.002			< 0.001
Single	13 (1.1)	2 (0.4)		41 (2.3)	3 (0.6)	
Married	1157 (97.8)	547 (96.3)		1547 (86.6)	306 (57.2)	
Divorced/widowed	13 (1.1)	19 (3.3)		199 (11.1)	226 (42.2)	
**Job status**			0.04			< 0.001
Employed	1157 (97.8)	547 (96.3)		320 (17.9)	139 (26.0)	
Unemployed	26 (2.2)	21 (3.7)		1467 (82.1)	396 (74.0)	
**Physical activity**			< 0.001			0.009
Low	536 (47.4)	171 (35.6)		516 (29.8)	171 (35.6)	
Moderate	337 (29.8)	199 (41.5)		980 (56.5)	199 (41.5)	
High	258 (22.8)	110 (22.9)		238 (13.7)	110 (22.9)	
**Current smoking**			< 0.001			0.09
Yes	339 (28.7)	82 (14.4)		69 (3.9)	13 (2.14)	
No	844 (71.3)	486 (85.6)		1718 (96.1)	522 (97.6)	
**Family history**			0.001			0.002
Yes	156 (13.2)	41 (7.2)		164 (9.2)	23 (4.3)	
No	1027 (86.8)	527 (92.8)		1623 (90.8)	512 (95.7)	

Values are expressed as mean ± SD for continuous variables and n (%) for categorical variables

The relationship between the physical activity pattern groups and demographic variables for men by age group are shown in Table 2. In men aged 40–60 years, there was a significant association between different physical activity pattern groups and smoking (*P* < 0.05). Also corresponding association were considered for woman in Table 3. A significant difference was observed between physical activity pattern groups and educational level in middle aged women, however, there were no significant associations between physical activity pattern groups and variables in woman aged > 60 years.

**Table 2:** Patterns of physical activity and its relationship with demographic variables for men based on age groups

Age groups	40–60				*P*-value	> 60				*P*-value
	Non-complier	Relapser	Adopter	Complier		Non-complier	Relapser	Adopter	Complier	
**Education**					0.53					0.25
Illiterate/primary	105 (38.7)	55 (37.2)	90 (34.0)	187 (41.8)		72 (84.7)	43 (86.0)	68 (79.1)	191 (73.7)	
Secondary/diploma	112 (41.3)	65 (43.9)	116 (43.8)	171 (38.3)		9 (10.6)	3 (6.0)	11 (12.8)	40 (15.4)	
Higher	54 (19.9)	28 (18.9)	59 (22.3)	89 (19.9)		4 (4.7)	4 (8.0)	7 (8.1)	28 (10.8)	
**Marital status**					0.16					0.39
Single	1 (0.4)	3 (2.0)	1 (0.4)	8 (1.8)		1 (1.2)	1 (2.0)	0 (0.0)	0 (0.0)	
Married	267 (98.5)	145 (98.0)	262 (98.9)	431 (96.4)		81 (95.3)	48 (96.0)	82 (95.3)	250 (96.5)	
Divorced/widowed	3 (1.1)	0 (0.0)	2 (0.8)	8 (1.8)		3 (3.5)	1 (2.0)	4 (4.7)	9 (3.5)	
**Job status**					0.65					0.98
Employed	264 (97.4)	143 (96.6)	261 (98.5)	437 (97.8)		81 (95.3)	48 (96.0)	83 (96.5)	248 (95.8)	
Unemployed	7 (2.6)	5 (3.4)	4 (1.5)	10 (2.2)		4 (4.7)	2 (4.0)	3 (3.5)	11 (4.2)	
**Smoking**					0.01					0.49
Yes	92 (33.9)	48 (32.4)	70 (26.4)	106 (23.7)		14 (16.5)	45 (90.0)	9 (10.5)	40 (15.4)	
No	179 (66.1)	100 (67.6)	195 (73.6)	341 (76.3)		71 (83.5)	5 (10.0)	77 (89.5)	219 (84.6)	
**Family history**					0.79					0.49
Yes	38 (14.0)	22 (14.9)	31 (11.7)	60 (13.4)		4 (4.7)	48 (96.0)	8 (9.3)	21 (8.1)	
No	233 (86.0)	126 (85.1)	234 (88.3)	387 (86.6)		81 (95.3)	2 (4.0)	78 (90.7)	238 (91.9)	

Values are expressed as mean ± SD for continuous variables and n (%) for categorical variables

Compliers: those who met the recommendations at both measurements

Non-compliers: those not reaching the recommended levels at both measurements

Adopters: those who were inadequately active at baseline but took up the recommendations at the second measurement

Relapsers: those who acted contrary to the adopter group

**Table 3:** Patterns of physical activity and its relationship with demographic variables for women based on age groups

Age groups	40–60				*P*-value	> 60				*P*-value
	Non-complier	Relapser	Adopter	Complier		Non-complier	Relapser	Adopter	Complier	
**Education**					0.006					0.11
Illiterate/primary	165 (74.0)	189 (71.3)	175 (59.9)	621 (65.2)		119 (99.2)	110 (98.2)	48 (94.1)	177 (95.2)	
Secondary/diploma	45 (20.2)	65 (24.5)	90 (30.8)	273 (28.6)		1 (0.8)	2 (1.8)	3 (5.9)	5 (2.7)	
Higher	13 (5.8)	11 (4.2)	27 (9.2)	59 (6.2)		0 (0.0)	0 (0.0)	0 (0.0)	4 (2.2)	
**Marital status**					0.82					0.45
Single	5 (2.2)	3 (1.1)	7 (2.4)	24 (2.5)		0 (0.0)	0 (0.0)	0 (0.0)	2 (1.1)	
Married	194 (87.0)	229 (86.4)	257 (88.0)	826 (86.7)		63 (52.5)	70 (62.5)	31 (60.8)	104 (55.9)	
Divorced/widowed	24 (10.8)	33 (12.5)	28 (9.6)	103 (10.8)		57 (47.5)	42 (37.5)	20 (39.2)	80 (43.0)	
**Job status**					0.11					0.47
Employed	29 (13.0)	47 (17.7)	62 (21.2)	172 (18.0)		28 (23.3)	26 (23.2)	13 (25.5)	56 (30.1)	
Unemployed	194 (0.87)	218 (82.3)	230 (78.8)	781 (82.0)		92 (76.7)	36 (76.8)	38 (74.5)	130 (69.9)	
**Smoking**					0.52					0.25
Yes	7 (3.1)	14 (5.3)	9 (3.1)	39 (4.1)		1 (0.8)	3 (2.7)	0 (0.0)	7 (3.8)	
No	216 (96.9)	251 (94.7)	283 (96.9)	914 (95.9)		119 (99.2)	109 (97.3)	51 (100.0)	179 (96.2)	
**Family history**					0.43					0.70
Yes	27 (12.1)	24 (9.1)	30 (10.3)	82 (8.6)		8 (6.7)	4 (3.6)	2 (3.9)	9 (4.8)	
No	196 (87.9)	241 (90.9)	262 (89.7)	871 (91.4)		112 (93.3)	108 (96.4)	49 (96.1)	177 (95.2)	

Values are expressed as mean ± SD for continuous variables and n (%) for categorical variables

Compliers: those who met the recommendations at both measurements

Non-compliers: those not reaching the recommended levels at both measurements

Adopters: those who were inadequately active at baseline but took up the recommendations at the second measurement

Relapsers: those who acted contrary to the adopter group

The results of Cox PH models for incidence of CVD and CHD for males and females are presented in Table 4 and Table 5, respectively. Unadjusted HR for CVD and CHD showed that compared to the reference group (non-compliers), in men aged 40–60 years, the incidence of CVD in the compliers and adopters were 0.55 (95%CI: 0.38–0.80, *P* = 0.002); 0.57 (95%CI: 0.38–0.87, *P* = 0.008) respectively; the corresponding values for the incidence of CHD were 0.57 (95%CI: 0.37–0.87, *P* = 0.01); 0.54 (95%CI: 0.37–0.79, *P* = 0.002), respectively (model 1). In men aged > 60 years, the incidence of CVD in adopters was 0.58 (95%CI: 0.36–0.93, *P* = 0.02). In middle-aged men, after adjusting for marital status, job status and education levels, HR for CVD incidence were 0.58 (95% CI: 0.36–0.91, *P* = 0.02) and 0.56 (95% CI: 0.37–0.84, *P* = 0.005) in the complier and adopter groups compared to non-compliers (model 2). The HR for CHD incidence of model 2 were 0.57 (95% CI: 0.35–0.92, *P* = 0.02) and 0.56 (95% CI: 0.37–0.86, *P* = 0.008) in the compliers and adopters, respectively. By including smoking status (model 3), HRs of CVD and CHD for middle-aged men in the complier group compared to the reference group were 0.58 (95% CI: 0.38–0.87, *P* = 0.009) and 0.58 (95% CI: 0.38–0.89, *P* = 0.01), respectively. The corresponding values for adopters compared to non-compliers were 0.60 (95% CI: 0.38–0.95, *P* = 0.03) and 0.59 (95% CI: 0.36–0.95, *P* = 0.03), respectively. Moreover in model 4, additional covariate family history of CVD or CHD was considered. HRs of CVD and CHD for men aged 40–60 years in the complier group were 0.58 (95% CI: 0.38–0.87, *P* = 0.008) and 0.58 (95% CI: 0.38–0.89, *P* = 0.01), respectively. HRs of CVD and CHD for men aged 40–60 years in adopter group were 0.61 (95% CI: 0.38–0.96, *P* = 0.03) and 0.60 (95% CI: 0.37–0.97, *P* = 0.04) respectively.

**Table 4:** Hazard ratios for development (incidence) of CVD and CHD based on age groups for men

Age groups	CVD				CHD			
	40–60		> 60		40–60		> 60	
	HR (95%CI)	*P*-value	HR (95%CI)	*P*-value	HR (95%CI)	*P*-value	HR (95%CI)	*P*-value
Model 1								
**Physical activity**								
Non-complier	Reference	_	Reference	_	Reference	_	Reference	_
Relapser	1.04 (0.69–1.59)	0.85	0.81 (0.49–1.33)	0.40	1.10 (0.71–1.67)	0.70	0.84 (0.49–1.45)	0.54
Adopter	0.57 (0.38–0.87)	0.008	0.58 (0.36–0.93)	0.02	0.57 (0.37–0.87)	0.01	0.64 (0.39–1.05)	0.08
Complier	0.55 (0.38–0.80)	0.002	0.73 (0.51–1.03)	0.07	0.54 (0.37–0.79)	0.002	0.73 (0.49–1.08)	0.11
Model 2								
**Physical activity**								
Non-complier	Reference	_	Reference	_	Reference	_	Reference	_
Relapser	1.07 (0.67–1.71)	0.95	0.90 (0.49–1.67)	0.75	1.11 (0.69–1.81)	0.65	0.93 (0.47–1.84)	0.82
Adopter	0.58 (0.36–0.91)	0.02	0.64 (0.37–1.13)	0.12	0.57 (0.35–0.92)	0.02	0.71 (0.39–1.32)	0.28
Complier	0.56 (0.37–0.84)	0.005	0.86 (0.55–1.33)	0.49	0.56 (0.37–0.86)	0.008	0.88 (0.54–1.45)	0.62
Model 3								
**Physical activity**								
Non-complier	Reference	_	Reference	_	Reference	_	Reference	_
Relapser	1.06 (0.66–1.70)	0.80	0.90 (0.49–1.67)	0.74	1.11 (0.69–1.80)	0.67	0.92 (0.46–1.82)	0.81
Adopter	0.60 (0.38–0.95)	0.03	0.65 (0.37–1.13)	0.13	0.59 (0.36–0.95)	0.03	0.74 (0.40–1.37)	0.33
Complier	0.58 (0.38–0.87)	0.009	0.86 (0.55–1.33)	0.49	0.58 (0.38–0.89)	0.01	0.89 (0.54–1.46)	0.64
Model 4								
**Physical activity**								
Non-complier	Reference	_	Reference	_	Reference	_	Reference	_
Relapser	1.05 (0.66–1.68)	0.84	0.90 (0.49–1.67)	0.74	1.11 (0.68–1.79)	0.68	0.92 (0.46–1.83)	0.81
Adopter	0.61 (0.38–0.96)	0.03	0.64 (0.37–1.13)	0.12	0.60 (0.37–0.97)	0.04	0.73 (0.39–1.35)	0.32
Complier	0.58 (0.38–0.87)	0.008	0.85 (0.55–1.32)	0.48	0.58 (0.38–0.89)	0.01	0.88 (0.54–1.44)	0.61

Hazard ratios (HR, 95% CI) for incidence of CVD and CHD calculated using extended Cox survival analysis

Model 1: Physical activity effect, unadjusted HR (95% CI) for CVD and CHD incidence

Model 2: Physical activity effect, adjusted for marital status, job status and education

Model 3: Physical activity effect, adjusted for marital status, job status, education and smoking

Model 4: Physical activity effect, adjusted for marital status, job status, education, smoking and family history of CVD or CHD

**Table 5:** Hazard ratios for development (incidence) of CVD and CHD based on age groups for women

Age groups	CVD				CHD			
	40–60		> 60		40–60		> 60	
	HR (95%CI)	*P*-value	HR (95%CI)	*P*-value	HR (95%CI)	*P*-value	HR (95%CI)	*P*-value
Model 1								
**Physical activity**								
Non-complier	Reference	_	Reference	_	Reference	_	Reference	_
Relapser	0.55 (0.33–0.92)	0.02	0.75 (0.49–1.15)	0.19	0.57 (0.33–0.98)	0.04	0.64 (0.39–1.04)	0.07
Adopter	0.43 (0.25–0.74)	0.002	0.56 (0.30–1.03)	0.06	0.47 (0.27–0.81)	0.007	0.57 (0.29–1.10)	0.09
Complier	0.48 (0.33–0.72)	< 0.001	0.73 (0.49–1.08)	0.11	0.52 (0.35–0.79)	0.002	0.77 (0.50–1.18)	0.23
Model 2								
**Physical activity**								
Non-complier	Reference	_	Reference	_	Reference	_	Reference	_
Relapser	0.92 (0.50–1.70)	0.80	0.84 (0.51–1.40)	0.51	0.88 (0.47–1.65)	0.69	0.77 (0.43–1.39)	0.39
Adopter	0.79 (0.42–1.49)	0.47	0.59 (0.28–1.25)	0.17	0.78 (0.40–1.50)	0.46	0.58 (0.25–1.35)	0.21
Complier	0.75 (0.45–1.25)	0.26	0.74 (0.46–1.19)	0.21	0.74 (0.44–1.26)	0.27	0.78 (0.46–1.33)	0.37
Model 3								
**Physical activity**								
Non-complier	Reference	_	Reference	_	Reference	_	Reference	_
Relapser	0.93 (0.51–1.72)	0.83	0.81 (0.49–1.35)	0.42	0.89 (0.47–1.68)	0.72	0.73 (0.41–1.31)	0.29
Adopter	0.80 (0.42–1.51)	0.49	0.63 (0.30–1.34)	0.23	0.79 (0.41–1.52)	0.48	0.64 (0.27–1.49)	0.30
Complier	0.76 (0.45–1.27)	0.29	0.72 (0.45–1.17)	0.19	0.76 (0.45–1.29)	0.30	0.76 (0.44–1.31)	0.32
Model 4								
**Physical activity**								
Non-complier	Reference	_	Reference	_	Reference	_	Reference	_
Relapser	0.95 (0.51–1.74)	0.86	0.82 (0.49–1.36)	0.44	0.90 (0.48–1.70)	0.75	0.74 (0.41–1.33)	0.31
Adopter	0.80 (0.42–1.51)	0.49	0.65 (0.30–1.37)	0.25	0.79 (0.41–1.51)	0.48	0.65 (0.28–1.53)	0.33
Complier	0.77 (0.46–1.29)	0.32	0.73 (0.45–1.19)	0.20	0.77 (0.45–1.31)	0.33	0.77 (0.45–1.32)	0.34

Hazard ratios (HR, 95% CI) for incidence of CVD and CHD calculated using extended Cox survival analysis

Model 1: Physical activity effect, unadjusted HR (95% CI) for CVD and CHD incidence

Model 2: Physical activity effect, adjusted for marital status, job status and education

Model 3: Physical activity effect, adjusted for marital status, job status, education and smoking

Model 4: Physical activity effect, adjusted for marital status, job status, education, smoking and family history of CVD or CHD

According to Table 5, the significant results are only observed in model 1 among middle aged women. Unadjusted HR for CVD and CHD showed that compared to reference category of physical activity (non-compliers), among study groups, in women aged 40–60 years, the incidence of CVD in the compliers, adopters and relapsers were 0.48 (95%CI: 0.33–0.72, *P* < 0.001), 0.43 (95%CI: 0.25–0.74, *P* = 0.002), and 0.55 (95%CI: 0.33–0.92, *P* = 0.02), respectively. The corresponding values for CHD were 0.52 (95%CI: 0.35–0.79, *P* = 0.002), 0.47 (95%CI: 0.27–0.81, *P* = 0.007), and 0.57 (95%CI: 0.33–0.98, *P* = 0.04), respectively.

## Discussion

The current longitudinal study investigates the relationship between physical activity patterns over time and incident cardiovascular outcomes in middle-aged and older men and women. Based on our results, middle-aged men who either constantly adhere to physical activity guidelines or adapt an active lifestyle over time have lower risk of developing CVD and CHD. These results were not significant for men in late adulthood and for women in both age groups.

In the current study more than half of the TLGS participants met the recommended levels of physical activity at baseline and follow-up prior to CVD incidence, respectively. Nevertheless, only 48.4% of our study population reported consistent adherence to the recommended levels of physical activity over the follow up period. National surveillance data also suggests that at least 40% of Iranian adults are inadequately active and this trend is increasing. In contrast to the national trend [12, 41], although prevalence of insufficient physical activity is still high, it shows a decreasing trend in the TLGS population. Further age-specific analysis in the current study showed that in mid-adulthood, insufficient physical activity is more frequent in men than women, while in elderly, the inactivity is more prevalent in women, making it an alarming health problem in older women residing in urban areas.

The current study demonstrated that compared to non-compliers, middle-aged men who maintained recommended levels of physical activity over time had the lowest CVD and CHD risk. Taking up the recommendations over time, was also associated with reduced CVD and CHD risk. Recently, few studies which have examined changes in physical activity, yielded the same results as ours, in middle-aged men [26, 27, 30]. There are also studies showing the same results in the total population, regardless of age and gender. Some studies have demonstrated that women could also benefit from sustained physical activity [28]. The data available on the elderly is less and controversial. Although questions remain about how much physical activity provides health benefits in the elderly, there are studies suggesting that physically active even less than recommended levels could be beneficial [42, 43]. Overall, the mechanisms underlying the effectiveness of exercise in preventing cardiovascular diseases are partially attributed to moderating the traditional cardio-vascular risk factors (hypertension, diabetes, and hyperlipidemia) [44]. However, PA mostly exerts its direct cardio-protective effects through its impact on endothelium and smooth muscle which prevents plaque formation and stabilizes the atherosclerotic lesions by improving vascular wall function [45–47]. These direct effects mostly appear through maintaining physical activity over time; therefore, sustained physical activity is needed to benefit from both short- and long-term protective effects of physical activity.

Our results show a gender-difference in the effects of physical activity changes on cardiovascular health. Existing literature is still controversial about the appropriate amount of physical activity (in terms of level, intensity, domain and pattern) to induce positive effects on the aging cardiovascular system in both genders [48]. Sex differences in normal cardiovascular aging are evident [49]; therefore the impact of physical activity on the aging cardiovascular system also appears to be sex-specific. This heterogeneity is not yet clearly explained, but one explanation could be the changes in sex hormones with age, baseline sex differences in cardiovascular system, and differences in the responsiveness of the cardiovascular system to physical activity [48, 49]. Another explanations for this gender disparities could be the different health-benefits attributed to various domains of PA; in particular, leisure time (LTPA) and occupational physical activity (OCPA), with the former being more cardiovascular protective [50]. Previous studies reported a decreasing trend in the LTPA domain in TLGS women over time [51]; this could partially elucidate why in our study women benefit less from their total physical activity over time. Another explanation could be additional effect of high compared to moderate physical activity. In their review Sattelmair, et al. [52] reported that, compared with those reaching the baseline recommendations, individuals who engaged in the advanced recommended level (300 min/week of moderate-intensity LTPA) had a 20% lower CVD/CHD risk. Among physically active Iranian men, a greater proportion of their activity is vigorous intensity activity compared to active Iranian women [41]. This may partly further explain the observed sex differences.

There could be another explanation for the sex differences seen in our results. A growing body of evidence indicates that sedentary lifestyle also plays a crucial role in attenuating chronic disease risks, especially in women [48]. Existing literature suggest that, although high levels of physical activity could eliminate the increased risk of death associated with high sitting time, other sedentary behaviors (in particular high screen time) could have independent negative effects on health outcomes that may not be fully eliminated by increasing physical activity [53]. Tracking physical activity profile of Iranian population shows that sedentary lifestyle is more prevalent in women than men in all ages, except in the eldest. Sedentary behaviors rise dramatically in men over 75 years [54]. This means that in order to obtain the health benefits of recommended amount of physical activity, individuals should be encouraged to reduce their sedentary habits as well. Although we saw the beneficial effects of an adaptive physical activity pattern in middle-aged men, our finding does not contradict the existing literature that the onset of physical activity -if accompanied by changes in other behaviors- can have positive effects at any age and in both sexes.

To the best of our knowledge, this is the first long-term population-based study of association between physical activity patterns and cardiovascular outcomes in Eastern-Mediterranean region and in Iran. The current analysis included a large sample size of middle-aged and older adults that made it possible for us to follow a relatively large population of older age for a long duration. Moreover, the PA was derived from main life domains i.e. leisure time and work-related in order to prevent underestimation. Nevertheless, the study has some limitations. First, subjective methods were used to measure PA which might increase the risk of recall bias and affect the accuracy of data. Second, in the TLGS, dietary measurements started from phase four; therefore diet was not included in the current study. Third, data on physical inactivity which also plays an effective role in cardiac health (e. g. time spent on sitting and watching TV) was not included in the current study; this may lead to the attenuation of this association. Fourth, as a part of TLGS, the current study was conducted in an urban area of Tehran; therefore the results may not be generalized to suburban and rural populations.

## Conclusions

In conclusion, both maintaining and up taking the recommended levels of physical activity over time would reduce the risk of incident CVD and CHD in middle-aged men. Our findings highlight the age- and gender-differences in this regard which requires further investigations, especially in the developing countries. Undoubtedly, adding physical activity components to lifestyle modifying programs has many health benefits, but it is of great importance to tailor them appropriately to meet the needs of any gender and age range.

## Data Availability

The datasets used and/or analyzed during the current study are available from the corresponding author on reasonable request.

## Abbreviations

CVD: Cardiovascular disease
CHD: Coronary heart disease
PA: Physical activity
TLGS: Tehran Lipid and Glucose Study
MAQ: Modifiable Activity Questionnaire
MET: Metabolic equivalent
HR: Hazard Ratio
CI: Confidence Interval
LTPA: Leisure-time physical activity
OCPA: Occupational physical activity
